# Gut metabolomics and 16S rRNA sequencing analysis of the effects of arecoline on non-alcoholic fatty liver disease in rats

**DOI:** 10.3389/fphar.2023.1132026

**Published:** 2023-03-27

**Authors:** Lingping Zhu, Duo Li, Xuefeng Yang

**Affiliations:** ^1^ Department of General Practice, Hengyang Medical School, The Affiliated Nanhua Hospital, University of South China, Hengyang, Hunan, China; ^2^ Hunan Provincial Clinical Research Center for Metabolic Associated Fatty Liver Disease, The Affiliated Nanhua Hospital, Hengyang Medical School, University of South China, Hengyang, Hunan, China

**Keywords:** arecoline, gut metabolites, 16S r RNA, NAFLD (non-alcoholic fatty liver disease), PGE2

## Abstract

**Introduction:** Non-alcoholic fatty liver disease (NAFLD) has gradually become the primary cause of fatty liver disease. Betel nuts have been used to treat gastrointestinal diseases.

**Methods:** In the present study, we analyzed the pathology, serology, gut flora, and metabolites in a rat model of NAFLD, with and without betel nut alkaloid treatment, using an integrated approach involving pathology, serological testing, 16S rRNA gene sequencing, and ultra-performance liquid chromatography-mass spectrometry metabolomics.

**Results:** Two rats were used for model validation. Thirty SD rats were included and divided into the normal group (C group), NAFLD model group (M group), low-dose group, medium-dose group (T group), and high-dose group with intraperitoneal injection of arecoline. The expression of blood lipids was significantly downregulated at all three arecoline concentrations (*p* < 0.05). Alpha-diversity analysis of the intestinal flora showed significant differences among the three groups, with a significant reduction in population diversity in the M group and a recovery of population diversity after arecoline treatment. At the phylum level, the relative abundance of *Firmicutes* was significantly higher in the T group and *Proteobacteria* in the M group. The KEGG metabolic pathways included polyketide sugar unit biosynthesis and hypertrophic cardiomyopathy. Thirty-three significantly different metabolites were identified among the groups. Significantly different metabolites between groups T and M included indolepyruvate, 2-deoxystreptamine, sakuranetin, glycyl-leucine, and riboflavin. The KEGG metabolic pathway suggested a potential role for arachidonic acid metabolism, serotonergic synapses, neuroactive ligand-receptor interactions, tyrosine metabolism, and regiomelanin. Vitamin digestion and absorption, as well as regulation of lipolysis in adipocytes, were the main metabolic pathways that distinguished the T vs. M groups. PGE2 is involved in several metabolic pathways. Correlation analysis showed that 29 bacterial species were significantly associated with PGE2 levels in the M and T groups. *Vagococcus*, *Lawsonia*, *Christensenella*, unidentified *Erysipelotrichaceae*, unidentified *Coriobacteriaceae*, and five other bacterial groups are unique in the PGE2 metabolic pathway regulated by arecoline.

**Discussion:** Arecoline has lipid-lowering effects and may exert therapeutic effects in NAFLD through intestinal metabolites and intestinal flora, as well as through the *Butyricicoccus*/*Christensenella*/*Coriobacteriaceae*-COX2/PGE2 pathway. Thus, arecoline may represent a potential drug or target for NAFLD treatment.

## 1 Introduction

Betel nut is a seed produced by the betel nut tree (Family Palmae), mainly in the Southern regions of Asia, such as in the Hainan and Yunnan Provinces, Taiwan, India, and Malaysia. Betel nuts for chewing are usually processed later and consist mainly of betel nut fruit, old-flowering vine, and calcined lime, with the addition of roasted tobacco and spices in some regions (IARC Working Group on the Evaluation of Carcinogenic Risks to Humans, 2004). Arecoline, arecaidine, guvacine, guvacoline, tannins, safrole, hydroxychavicol, and catechins are components of betel nuts (Tilakaratne et al., 2006). Betel nuts have historically been used as a proprietary Chinese medicine for the treatment of a variety of ailments. Recent research has found that they have anthelmintic, neuroprotective, digestive, antidepressant, cardiovascular, antiinflammatory, and antiviral effects (Kim et al., 2018). Moreover, betel nuts inhibited cholesterol absorption. In addition, the blood lipid levels, small intestine pancreatic cholesterol esterase activity, and intestinal cholesterol acyltransferase activity of rats, which were fed 10% triglycerides and betel nut extract, were lower than those of the control group without betel nut extract. The absorption of triglycerides decreased with supplementation of the betel nut extract, indicating that the inhibition of pancreatic cholesterol esterase may have inhibited pancreatic lipase activity, resulting in lower plasma triglyceride levels (Byun et al., 2001).

Non-alcoholic fatty liver disease (NAFLD) is primarily caused by hepatocellular steatosis, a manifestation of excessive accumulation of toxic lipids, such as triglycerides, free fatty acids (FAA), ceramides, and free cholesterol in the liver (Marra and Svegliati-Baroni, 2018). The current symptoms of NAFLD include overweight/obesity, metabolic dysfunction, and diabetes with metabolic dysfunction including high triglyceride levels (Eslam et al., 2020) and its role in the development of NAFLD. Altered intestinal flora is an important factor in the development of NAFLD, including small intestinal bacterial overgrowth (Wigg et al., 2001), alterations in the intestinal flora metabolite TMAVA (Zhao et al., 2020), production of endogenous acetaldehyde (Cope et al., 2000), and increased intestinal permeability (Miele et al., 2009), which also play a role in the reversal of NAFLD. After 4 weeks of probiotic administration, remarkable improvement was observed in the symptoms of NAFLD mice with fatty liver-related indices (Li et al., 2010).

However, the role of arecoline in NAFLD remains unclear. A study from Taiwan showed that metabolic syndrome increased the risk of substantial liver fibrosis, and cumulative betel nut exposure increased the risk of substantial liver fibrosis in patients with metabolic syndrome, but not in those without metabolic syndrome (Chou et al., 2022). However, a previous study suggested a hypolipidemic effect of arecoline (Byun et al., 2001). Given that the current study remains controversial, we constructed NAFLD rats and explored the effects of arecoline on NAFLD-induced pathophysiological mechanisms with respect to lipid levels, pathology, 16S rRNA sequencing, and metabolomics.

We used a total of 32 rats, two of which were used to validate the NAFLD model; six healthy controls and six rats from each NAFLD model were treated with low, medium, and high arecoline concentrations. Total DNA was extracted from fresh feces of the healthy control, model, and medium-concentration treatment groups, and 16S rRNA gene sequencing and metabolic analysis were performed using ultra-performance liquid chromatography-mass spectrometry (UHPLC-MS). The DNA was analyzed using UHPLC-MS for 16S rRNA gene sequencing and metabolic profiling. We found differences in the intestinal flora and metabolites, which may be of value as the cause of NAFLD and candidate biomarkers for early diagnosis and treatment.

## 2 Materials and methods

### 2.1 Mice breeding and NASH modeling

Thirty-two male SD rats (3 weeks old) of the SFP class were housed for 1 week of acclimatization and randomly divided into two groups: one group (seven rats) on a conventional diet and the other group (25 rats) on a high-fat diet. After 4 weeks of consuming the high-fat diet, the rats in the high-fat diet group were intraperitoneally injected with 30 mg/kg of STZ (pre-formulated with citrate buffer, 30 mg/mL; fasting blood glucose levels were measured 3 days after injection). After 5 weeks of feeding on high-fat chow, blood was collected from all rats into sterile centrifuge tubes, left at room temperature for 2 h, centrifuged at 4°C for 15 min at 3,000 rpm, and the supernatant was removed for immediate measurement of lipids (HDL-C, TC, TG, and LDL). One rat in the conventional diet group and one rat in the high-fat diet group were sacrificed, and their livers were fixed in 4% paraformaldehyde solution, embedded in paraffin, sectioned, and stained with heatoxylin and eosin (HE) to observe the lesions. After 6 weeks of feeding on a high-fat diet, the rats in the high-fat diet group were randomly divided into four groups: high-fat model control group and low-, medium-, and high-dose groups with intraperitoneal injection of arecoline of concentrations 0.5, 1, and 5 mg/kg, respectively, for 4 weeks. On the day after drug withdrawal, blood was collected from all rats in sterile centrifuge tubes, incubated at room temperature for 2 h, and then centrifuged at 3,000 rpm for 15 min at 4°C. The supernatant was collected for determination of lipids (HDL-C, TC, TG, and LDL). Feces were collected from all rats and stored at −80°C for subsequent 16s rRNA sequencing and metabolomic sequencing. Rats were sacrificed, and their livers were divided into two groups: one group was fixed in 4% paraformaldehyde solution and the other was stored at −80°C. Blood specimens were collected from the rats and tested for HDL-C, TC, TG, and LDL levels using a fully automated biochemical instrument. The livers were sectioned and stained with HE.

### 2.2 Fecal 16S rRNA sequencing analysis

DNA was quantified using a Nanodrop, and the quality of the extracted DNA was checked using 1.2% agarose gel electrophoresis. PCR amplification of the variable regions of the rRNA gene (single or continuous) or specific gene fragments was performed by adding sample-specific barcode sequences to primers designed according to the conserved regions of the sequence. Magnetic beads (Vazyme VAHTSTM DNA clean beads) of × 0.8 volume of the PCR product were added to 25 μL of the PCR product. The recovered PCR amplification products were quantified by fluorescence using a Quant-iT PicoGreen dsDNA assay kit and microplate reader (BioTek, FLx800). Based on the fluorescence quantification results, each sample was mixed in an appropriate ratio according to the amount of sequencing required for each sample. Sequencing libraries were prepared using an Illumina TruSeq Nano DNA LT Library Prep Kit. The amplification products were first repaired by removing the prominent base at the 5ʹ end of the DNA sequence, adding a phosphate group to complete the missing base at the 3ʹ end, adding an A base at the 3ʹ end of the DNA sequence to prevent self-association of the DNA fragment and to ensure that the target sequence could be attached to the sequencing junction (a prominent T base at the 3ʹ end of the sequencing junction), and adding a sequencing junction containing a library-specific tag (i.e., index sequence) to the 5ʹ end of the sequence to enable the DNA molecule to be immobilized on the flow cell. BECKMAN AMPure XP beads were used to purify the library system after the addition of the junction by removing self-associated fragments through magnetic bead screening. PCR amplification was performed on the junctioned DNA fragments to enrich the sequencing library template, and BECKMAN AMPure XP beads were used to purify the library enrichment products. The final selection and purification of the library were performed using 2% agarose gel electrophoresis. Before sequencing, the libraries were quality-checked on an Agilent Bioanalyzer using an Agilent High-Sensitivity DNA kit. The qualified libraries had a single peak and were free of junctions. The libraries were quantified on a Promega QuantiFluor fluorescence quantification system using the Quant-iT PicoGreen dsDNA assay kit. The qualified libraries should have a concentration of 2 nM or more. Raw downstream data from high-throughput sequencing were initially screened according to sequence quality, and problematic samples were retested and replated. The raw sequences that passed the initial quality screening were divided into libraries and samples according to index and barcode information, and the barcode sequences were removed. Sequence denoising or OTU clustering was performed according to the analysis process of the QIIME2 dada2 or Vsearch software. The specific composition of each sample (group) at different taxonomic levels is presented as an overview. The alpha-diversity level of each sample was assessed based on the distribution of ASV/OTU across different samples, and the appropriateness of the sequencing depth was reflected by the sparsity curve. At the ASV/OTU level, the distance matrix of each sample was calculated, and the difference in beta diversity between different samples (groups) and the significance of the difference was measured using a variety of unsupervised ranking and clustering means, combined with the appropriate statistical tests. At the species taxonomic composition level, differences in species abundance composition between samples (groups) were further measured using various unsupervised and supervised ranking, clustering, and modeling instruments, combined with corresponding statistical tests, and attempts were made to identify marker species. Based on the distribution of the species composition across samples, association networks were constructed, topological indices were calculated, and attempts were made to identify key species. Based on the 16S rRNA, 18S rRNA, and ITS gene sequencing results, it was also possible to predict the metabolic function of the sample flora, identify differential pathways, and obtain the species composition of specific pathways.

### 2.3 Analysis of gut LC-MS metabolites

The raw data were first converted to the mzXML format using MSConvert in the ProteoWizard software package (v3.0.8789) (Yu et al., 2006) and processed using XCMS (Manish et al., 2007) for feature detection, retention time correction, and alignment. The metabolites were identified using accuracy mass (<30 ppm) and MS/MS data that were matched with HMDB (Abdelrazig et al., 2020) (http://www.hmdb.ca), massbank (Gagnebin et al., 2017) (http://www.massbank.jp/), LipidMaps (Thévenot et al., 2015) (http://www.lipidmaps.org), mzcloud (Xia and Wishart, 2011) (https://www.mzcloud.org), and KEGG (Dunn et al., 2011) (http://www.genome.jp/kegg/). Robust LOESS signal correction (QC-RLSC) (Want et al., 2013) was applied for data normalization to correct for any systematic bias. After normalization, only ion peaks with relative standard deviations (RSDs) less than 30% in QC were retained to ensure proper metabolite identification.

The Ropls (Anne-Laure and Korbinian, 2007) software was used for all multivariate data analyses and modelling. The data were mean-centered using scaling. The models were built using principal component analysis (PCA), orthogonal partial least squares discriminant analysis (PLS-DA), and partial least squares discriminant analysis (OPLS-DA). Metabolic profiles can be visualized as a score plot, where each point represents a sample. Corresponding loading plots and S-plots were generated to provide information on the metabolites that influenced the clustering of the samples. All evaluated models were tested for overfitting using permutation tests. The descriptive performance of the models was determined using R2X (cumulative) [perfect model: R2X (cum) = 1] and R2Y (cumulative) [perfect model: R2Y (cum) = 1] values, whereas their prediction performance was measured using Q2 (cumulative) [perfect model: Q2 (cum) = 1] and a permutation test. The permuted model should not be able to predict classes; R2 and Q2 values at the *Y*-axis intercept must be lower than those of Q2 and R2 of the non-permuted model. OPLS-DA allowed the determination of metabolites using variable importance on projection (VIP). The *p*-value, VIP produced by OPLS-DA, and fold change (FC) were applied to discover the variable contributing to classification. Finally, *p*-values <0.05, and VIP values >1 were considered significant. Differential metabolites were subjected to pathway analysis using MetaboAnalyst (Trygg and Wold, 2010), which combines results from powerful pathway enrichment analysis with pathway topology analysis. The metabolites identified using metabolomics were then mapped to the KEGG pathway for biological interpretation of higher-level systemic functions. Metabolites and their corresponding pathways were visualized using the KEGG Mapper tool. Spearman’s correlation coefficients were used to analyze the relationship between metabolomics and the gut microbiota, and the ROC curve was used to explore potential biomarkers.

### 2.4 Statistical analysis

GraphPad prism 5 (GraphPad Software, San Diego, CA) was used for statistical analysis; *p* < 0.05 and *p* < 0.01 were used as screening criteria for significant and highly significant differences, respectively.

## 3 Results

1. Clinical and pathological information

**FIGURE 1:**
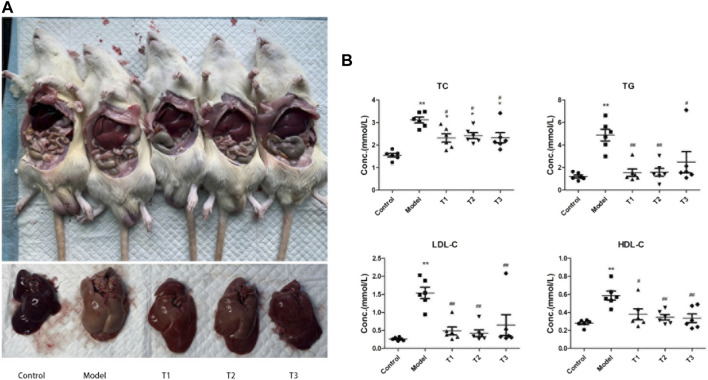
**(A)** Gross liver map of mice from each group; **(B)** Lipid levels, * and ** indicates significant (*p* < 0.05) and highly significant (*p* < 0.01) difference compared with group C, respectively; # and ## indicates significant (*p* < 0.05) and highly significant (*p* < 0.01) difference compared with group M, respectively; T1: mice injected with low concentration of arecoline, T2: mice injected with medium concentration of arecoline, and T3: mice injected with high concentration of arecoline.

Successful modeling was demonstrated using pathological findings and serum lipid levels in the healthy (C group) and the NAFLD model groups (M group). The results of lipid testing showed that the levels of total cholesterol (TC), triglycerides (TG), low-density lipoprotein cholesterol (LDL-C), and high-density lipoprotein cholesterol (HDL-C) were increased in the M group. TC, LDL-C, and TG levels were significantly increased (*p* < 0.05, for TC and LDL-C; *p* < 0.01, for TG) (Supplementary Figure S1). After arecoline treatment, significant differences were detected between the experimental groups. The levels of TC, LDL-C, and HDL-C were significantly increased in the M group (*p* < 0.01), and all three arecoline concentrations significantly decreased the levels of TC, TG, LDL-C, and HDL-C (*p* < 0.01) (Figure 1).

2. Sequencing depth and diversity analysis of intestinal microbiota

**FIGURE 2:**
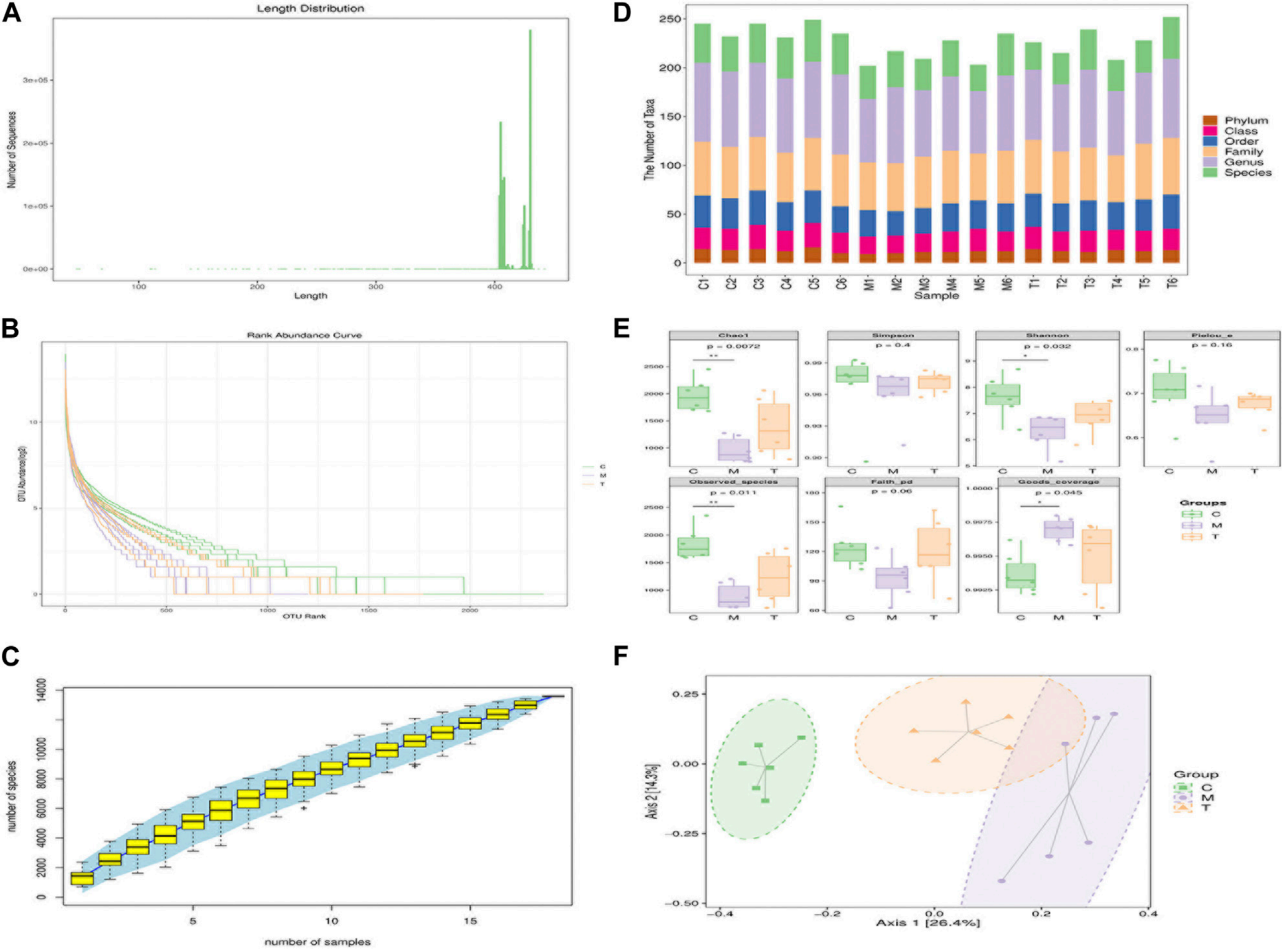
**(A)** Sequence length distribution of 16s RNA post-sequencing; **(B)** Sequencing abundance rank curve. The horizontal coordinate represents the ordinal number of ASVs/OTUs in order of abundance; the vertical coordinate represents log2 log value of the abundance (mean) of each ASV/OTU in the sample (group); each fold line represents a sample (group) and the length of the fold line on the horizontal axis reflects the abundance of that group in terms of ASV/OTU number. **(C)** Specaccum species accumulation curve plot. The horizontal coordinate represents the sample size, the vertical coordinate represents the number of species (ASV/OTU) observed, and the blue shaded region reflects the confidence interval of the curve. The results reflect the rate of increase in the number of new species observed as the sample size expanded over the course of sampling. **(D)** Statistics of the number of microbial taxonomic units observed at each level. The horizontal coordinates represent the sample names and the vertical coordinates represent the number of taxonomic units contained at each of the six levels of phylum, order, family, genus and species. **(E)** Box plot of the groupings of α diversity. The horizontal coordinates represent the grouping labels and the vertical coordinates represent the values of the corresponding α diversity indices. **(F)** Principal Coordinates Analysis (PCoA); Two-dimensional ranking plot of the samples.

We used 16S rRNA sequencing to analyze the gut microbiota profiles of rats in the C, M, and T groups (arecoline treatment group). We observed the total gut microbiota with fragment lengths above 400 bp (Figure 2A), and high abundance and homogeneity (Figure 2B), indicating that we could obtain sufficient sequencing information from samples from the three groups. *Specaccum* species accumulation plots reflected a progressively lower rate of the increase of new species observed with continued sample size expansion, indicating that we had a sufficient sample size. Figure 2D shows the number of taxonomic units at each taxonomic level for the different samples; the M group had significantly fewer taxonomic units than the other two groups, indicating that the taxonomic richness of the M group was less than that of the other two groups. Chao1, observed species, Shannon, Simpson, Faith’s phylogenetic diversity, and Pielou’s evenness indices decreased in the M group, indicating that the M group was poorer than the other two groups in terms of richness and diversity. Moreover, the increase in the Good’s coverage index was most obvious in the M group. On the other hand, all three groups had >99% sequence coverage, which indicates that the sequence coverage was adequate (Figure 2E). The PCA score analysis showed that the three groups were well-distributed among the different groups (Figure 2F), with some overlap between the M and T groups. The composition of the top 20 species of different genera is presented as a histogram (Supplementary Figure S2).

3. Analysis of the composition of intestinal microorganisms

**FIGURE 3:**
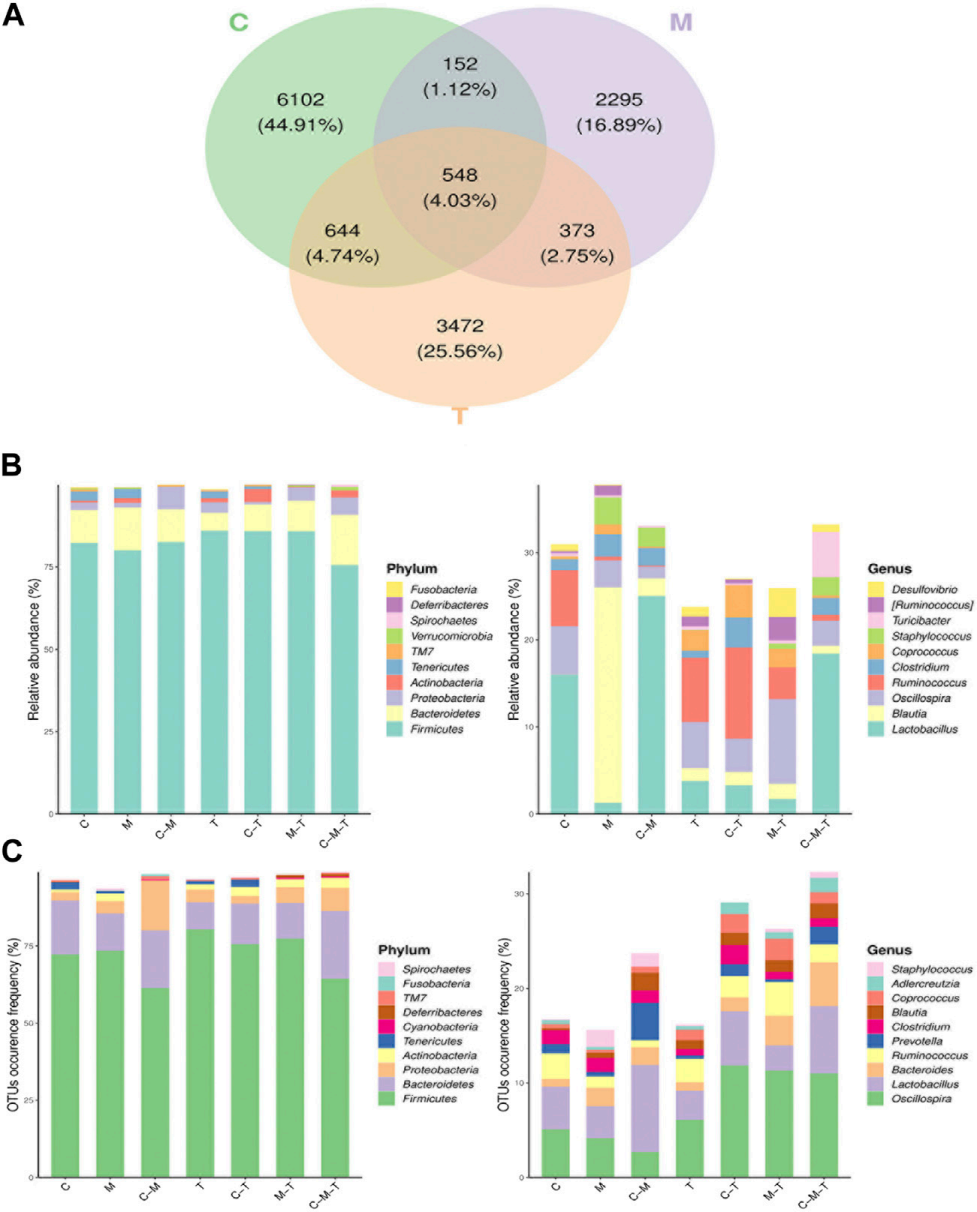
**(A)** Venn diagram of ASV/OTU of the groups. The overlapping areas between ellipses indicate the common ASV/OTU among the samples (groups) and the number in each block indicates the number of ASV/OTU contained in that block; **(B)** Histogram of ASV/OTU abundance in different regions of the Venn diagram. The horizontal coordinates represent the sets of ASV/OTU corresponding to different regions of the Venn diagram and the vertical coordinates represent the ASV/OTU belonging to different clades. The top 10 phyla and genera with the highest average sequence abundance in the sample are shown; **(C)** Histogram of the number of ASV/OTU in different regions of the Venn diagram. The horizontal coordinate represents the set of ASV/OTU corresponding to different regions of the Venn diagram and the vertical coordinate represent the percentage of ASV/OTU belonging to different phyla. The top 10 classes and genera with the highest average ASV/OTU frequency are also shown.

Community analysis was performed using a Venn diagram, in which 6,102 OTUs were unique to the C group, 700 OTUs overlapped in the C and M groups, 1,192 OTUs overlapped in the C and T groups, and 921 OTUs overlapped in the T and M groups, for a total of 548 OTUs in the three groups (Figure 3A). Analysis of the relative abundance of each group in the Venn diagram at the phylum level revealed that the relative abundances of *Firmicutes* and *Proteobacteria* were significantly higher in the T and M groups, respectively. At the genus level, the relative abundances of *Lactobacillus* and *Ruminococcus* declined, whereas those of *Staphylococcus*, *Blautia*, and *Clostridium* were increased in the M group (Figure 3B). In contrast, the relative abundance of *Lactobacillus*, *Oscillospira*, and *Ruminococcus* was higher in the T group than in the C group. In terms of the frequency of OTU occurrence, the proportion of *Oscillospira*, *Bacteroides*, and *Prevotella* increased in each group at the genus level (Figure 3C).

4. Screening for differentially enriched gut flora caused by arecoline treatment

**FIGURE 4:**
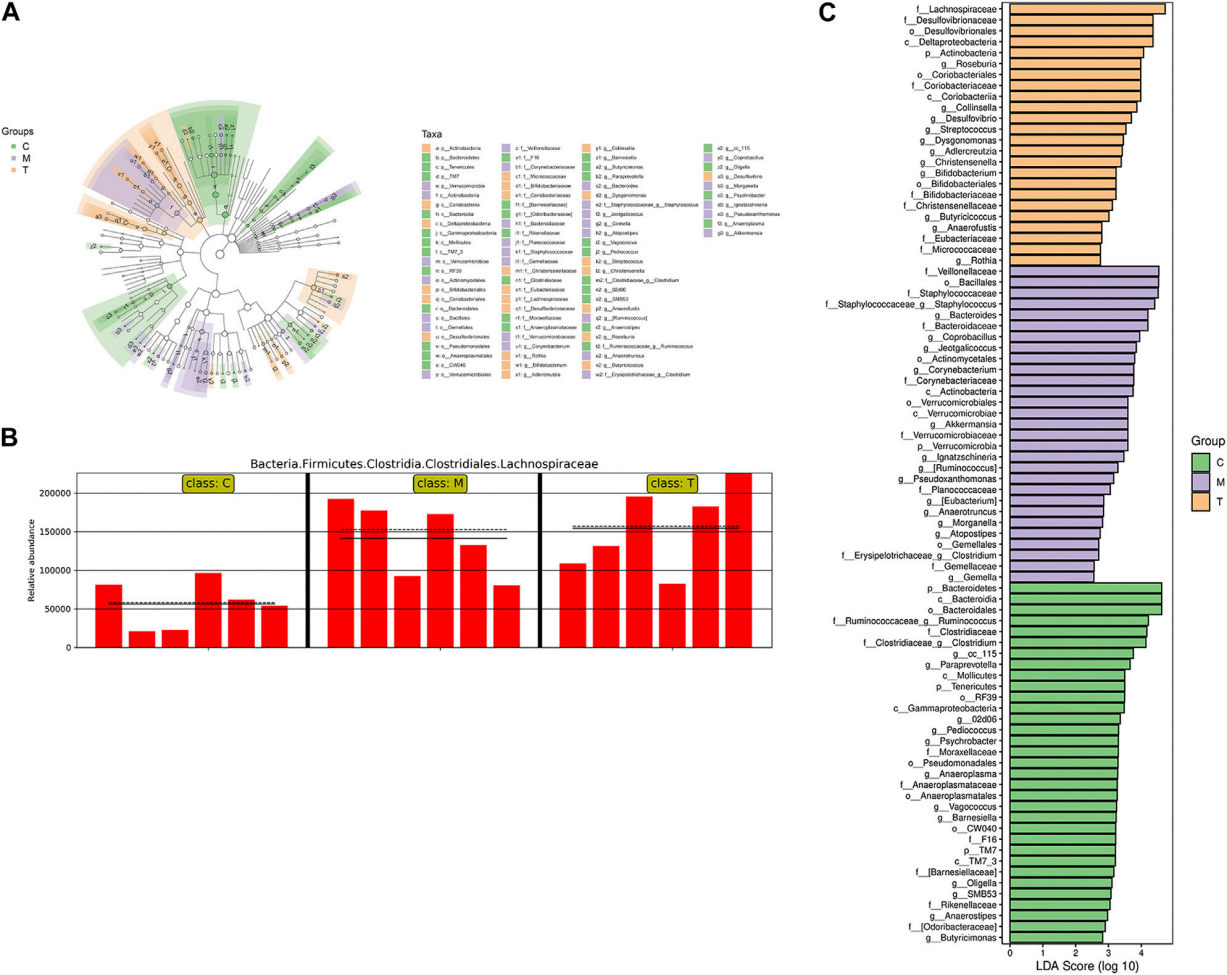
**(A)** Taxonomic hierarchy tree of the between-group differences in the taxonomic units. The circles from inside to the outside of the branching diagram represent the taxonomic levels from phylum to species, with the diameter of each circle proportionate to the relative abundance of the gut microbiota. Node size corresponds to the average relative abundance of that taxon; hollow nodes represent taxa that do not differ significantly between groups, while nodes in other colors indicate that these taxa show significant between-group differences and are more abundant in the subgroup sample represented by that color. Letters represent the names of taxa with significant inter-group differences. **(B)** Relative abundance of Lachnospiraceae in the three groups; the solid and dashed lines represent the mean and median relative abundance of the taxon in each group, respectively. **(C)** Histogram of Linear discriminant analysis (LDA) effect values for marker species. The vertical coordinates represent the taxa that differ significantly between groups, while the horizontal coordinates represent the logarithmic LDA scores for each taxon. The categorical units are ranked according to their scores, which describes their specificity within the sample group. The longer the length, the more significant the difference between the taxa, where the color of the bars indicates the sample group with the highest abundance for that taxon.

Heat maps of single- and two-level species clustering were used to demonstrate differences in species at the genus level (Supplementary Figures S3 A, B). LEfSe (LDA effect size) analysis combines non-parametric Kruskal–Wallis and Wilcoxon rank sum tests with linear discriminant analysis (LDA) effect size. In the branching plots and histograms in Figures 4A, C, the circles from the inside to the outside of the branching plots represent the taxonomic level from phylum to species, and the diameter of each small circle is proportional to the relative abundance of the gut microbiota. The letters “p,” “c,” “o,” “f,” “g,” and “s” represent the phylum, order, family, genus, and species, respectively. At the taxonomic level, from phylum to species, three significantly different components of the gut flora were identified among the three groups (LDA score ≥2.5): in group T, *f_Lachnospiraceae*, *f_Desulfovibrionales*, *o_Desulfovibrionaceae*, *c_Deltaproteobacteria*, and *p_Actinobacteria*; and in group M, *f_Veillonellaceae*, *o_Bacillales*, and *f_Stacillales*. Bacillales, *f_Staphylococcaceae*, *f_Staphylococcaceae*, *g_Staphylococcus*, *g_Bacteroides*, and *f_Bacteroidaceae* from group M, and *p_Bacteroidetes*, *c_Bacteroidales*, and *o_Bacteroidales* from C group. Bacteroidales, *o_Bacteroidia*, *f_Ruminococcaceae*_*g_Ruminococcus*, *f_Clostridiaceae*, and *f_Clostridiaceae*_*g_Clostridium*, all of which had an LDA ≥4. In addition, Lanchnospiraceae is shown in a bar chart (Figure 4B), indicating that it was the most significantly different group from the other two in the T group.

5. Pathways analysis of differentially abundant gut microbiota

**FIGURE 5:**
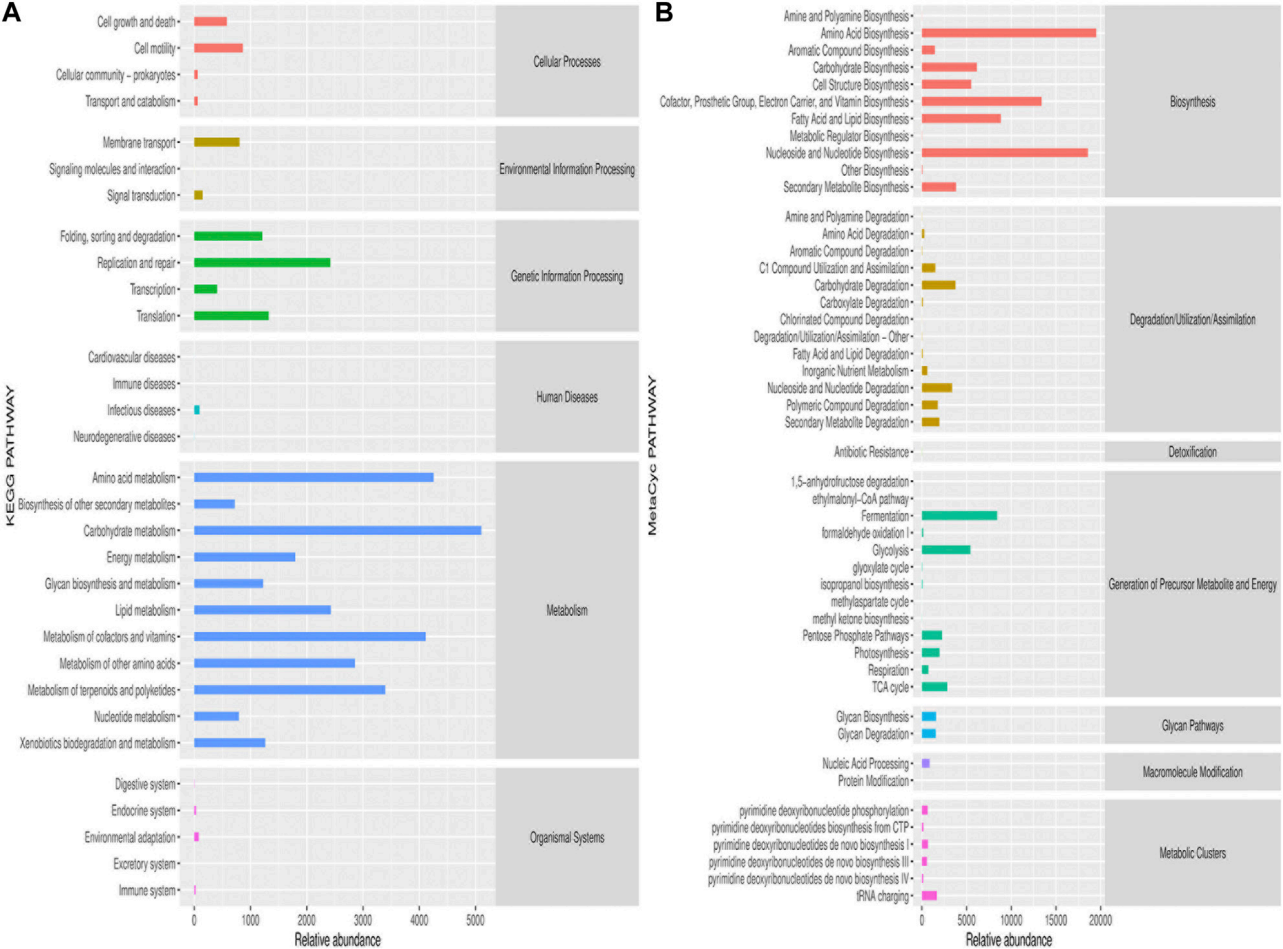
**(A)** Predicted abundance of KEGG secondary functional pathways. The horizontal coordinates represent the abundance of functional pathways (per million KOs), the vertical coordinates represent the functional pathways at the second KEGG classification level, and the rightmost column represent the first level pathways to which these pathways belong. The average abundance of all the samples is shown. **(B)** Predicted abundance of MetaCyc secondary functional pathways. The horizontal coordinate represents the abundance of the functional pathway (per million KO), the vertical coordinate represents the functional pathway at the second KEGG classification level, and the rightmost column represents the first level pathways to which these pathways belong. The average abundance of all the samples is shown.

We then predicted the pathways and metabolic processes using the KEGG Pathway Database (http://www.genome.jp/kegg/pathway.html) and MetaCyc pathways (https://metacyc.org/) for significantly different gut flora. The main enrichment pathway of KEGG was the metabolism of amino acids (Figure 5A), whereas the main pathways enriched by the MetaCyc included amino acid biosynthesis, nucleoside and nucleotide biosynthesis, cofactor, prosthetic group, electron carrier, vitamin biosynthesis, fermentation, and glycolysis (Figure 5B). A dominant species seed network map with grouped abundance pie charts was used to show the interconnections of the differential groups and the proportion of each group in the differential group (Supplementary Figure S4A). In addition, the KEGG pathway analysis showed differences between groups with respect to metagenomeSeq, polyketide sugar unit biosynthesis, and hypertrophic cardiomyopathy (HCM) (Supplementary Figure S4B). PWY-6572 (chondroitin sulfate degradation I (bacterial)) was the MetaCyc metabolic pathway with the most significant difference between the groups (Supplementary Figure S4C). The LEU-DEG2-PWY (L-leucine degradation I) of the differential species composition was used to compare the MetaCyc pathway between groups (Supplementary Figure S4D).

6. Multivariate analysis of fecal metabolite profiles

**FIGURE 6:**
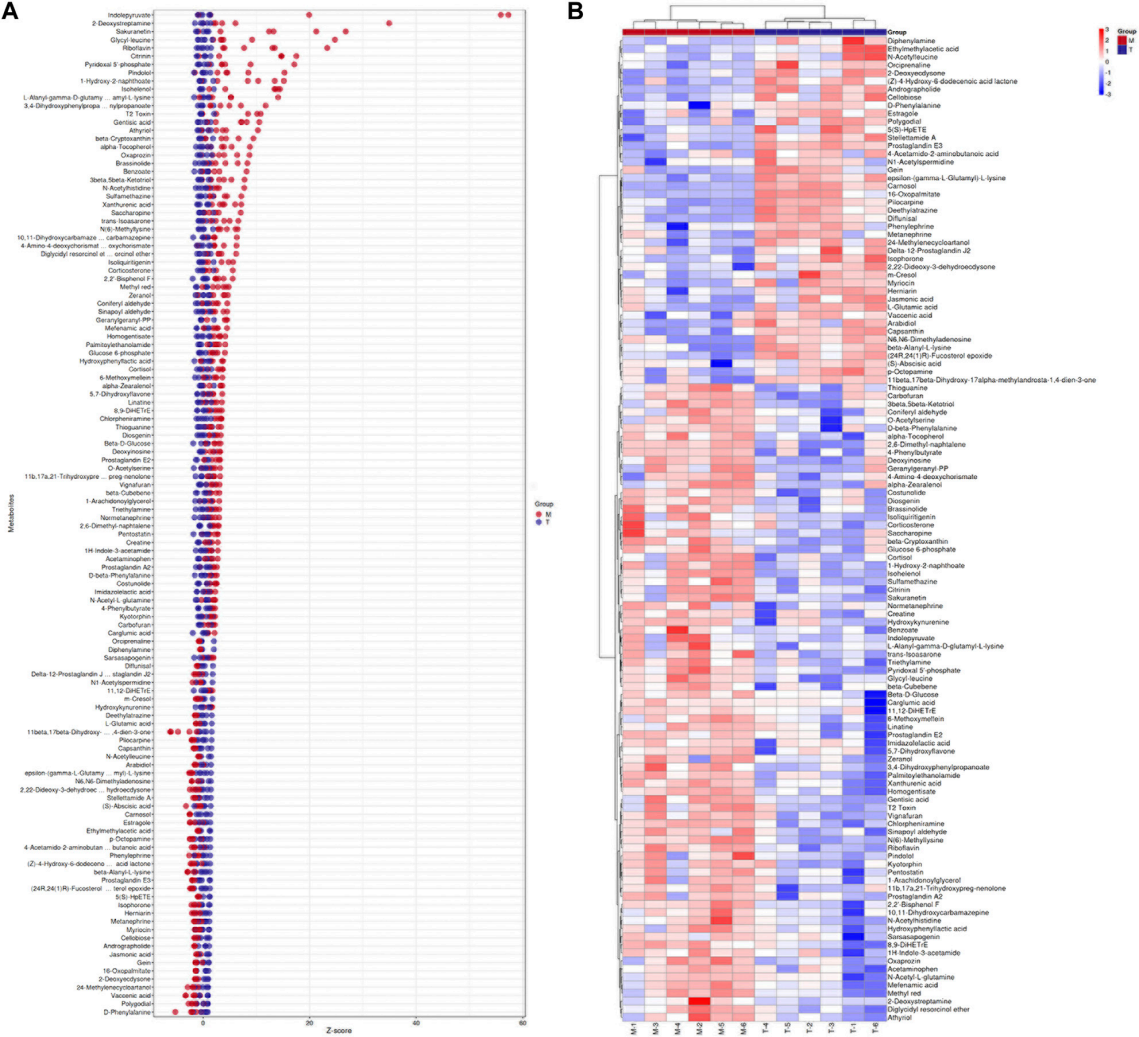
**(A)** Z-score plot (T vs. M group), the vertical coordinate represents the name of the metabolite, the color of the dots represent the different groups, the horizontal coordinate represents the relative content of the metabolite in the group obtained using the Z-score, the more it is towards the right, the more is the metabolite in the group. **(B)**. Hierarchical clustering of differential metabolites. The columns represent groups and the rows represent metabolite names. The differential metabolite clustering tree is shown on the left side of the graph.

The orthogonal projection potential structure discriminant analysis (OPLS-DA) model was obtained from UHPLC-MS, and the profiles of the three groups of samples were separated using positive ion mode (POS) and negative ion mode (NEG). The heat maps of the three groups are shown in Supplementary Figure S5C. The three groups can be more clearly distinguished, and the heat map in Supplementary Figure S6A can also more clearly distinguish the T and M groups. We then screened out the differential metabolites between the T and M groups and illustrated them using a volcano plot, as shown in Supplementary Figure S6B, where the red dots indicate upregulation of metabolite expression in the T group compared to that in the M group, whereas the blue dots indicate downregulation of metabolite expression in the T group. This finding suggests that arecoline treatment in the NAFLD group triggered substantial changes in metabolite levels. To visually show the differences in metabolites between the three groups, we used the PLS-DA-Score Plot for display (Supplementary Figure S5A), along with a Venn diagram and bar graphs to show the metabolites shared and grouped between the three groups (Supplementary Figures S5B, D), along with a Z-score (standard score) plot to show the difference in relative content values between the two groups (Figure 6A), whereas the MS/MS heat map enabled the clustering and identification of metabolites in which significant differences were detected, with indolepyruvate, 2-deoxystreptamine, sakuranetin, glycyl-leucine, riboflavin, and citrinin showing the most significant differences between the T and M groups (Figure 6B).

7. Pathway analysis of differentially enriched metabolites

**FIGURE 7:**
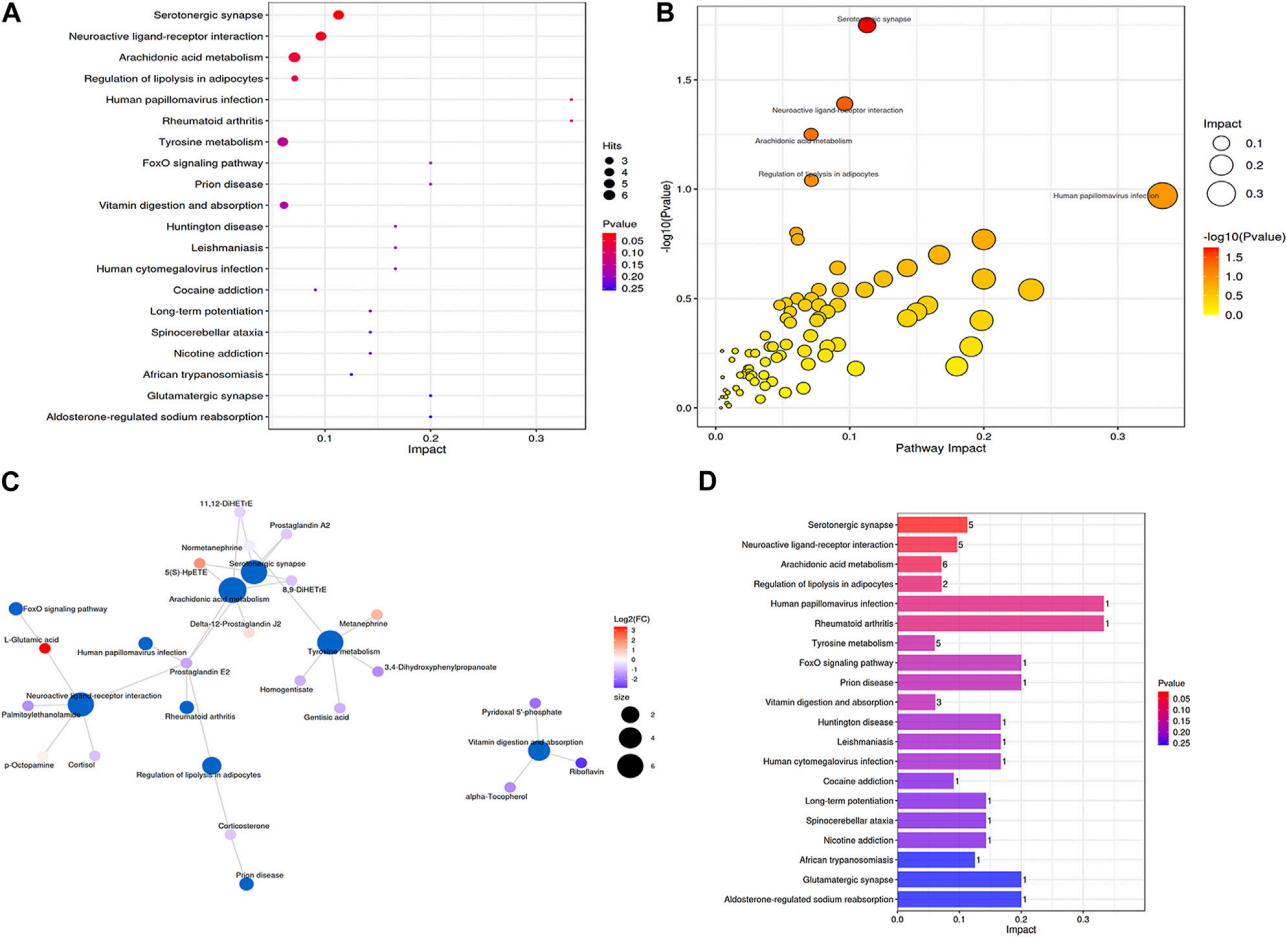
**(A)** Metabolic pathway impact factor bubble diagram 1; **(B)** Metabolic pathway impact factor bubble diagram 2. Small *p*-value represents more significant effect of the detected differential metabolite on the pathway. The larger the impact value, the greater the contribution; **(C)** Metabolite and metabolic pathway network diagram; **(D)** Metabolic pathway impact factor histogram. The vertical coordinates represent the metabolic pathways and the horizontal coordinates represent the enrichment of different metabolic pathways Impact values.

The results from the Metabolic Analyst website (https://www.metaboanalyst) are presented as bubble plots of metabolic pathway impact factors, as shown in Figure 7A. Arachidonic acid metabolism, serotonergic synapses, neuroactive ligand-receptor interactions, tyrosine metabolism, vitamin digestion and absorption, and regulation of lipolysis in adipocytes were the metabolic pathways that distinguished the T and M groups (Figures 7A, B). The first three pathways are the main metabolic pathways. Delta-12-prostaglandin J2, 5(S)-HpETE, 8,9-DiHETrE, prostaglandin E2, 11,12-DiHETrE, 8,9-DiHETrE, and prostaglandin A2 were enriched in this metabolic pathway. Moreover, arachidonic acid metabolism, 11,12-DiHETrE, normetanephrine, 8,9-DiHETrE, prostaglandin A2, and 5(S)-HpETE were enriched in the metabolic pathway. Serotonergic synapses, L-glutamic acid, palmitoylethanolamide, p-octopamine, cortisol, and prostaglandin E2 were enriched in the neuroactive ligand-receptor interaction (Figure 7C). The present study indicates that prostaglandin E2 plays a role in a variety of metabolic pathways. We also ranked the contribution and *p*-values and found that the serotonergic synapse had the highest contribution and lowest *p*-value, indicating that it is an important pathway for the therapeutic effects of arecoline (Figure 7D).

8. Correlation analysis of gut microbes and metabolites

**FIGURE 8:**
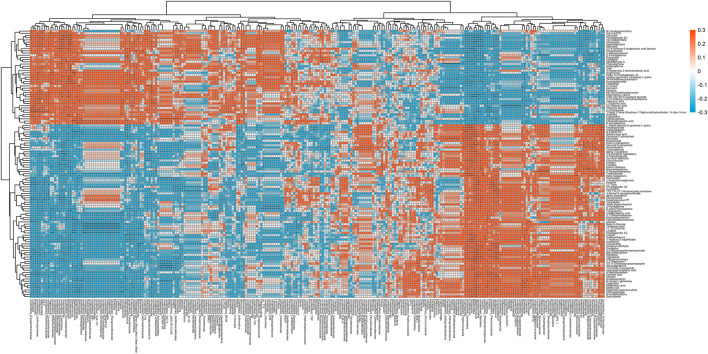
Heat map of significantly different genus levels and significantly different metabolites in T vs. M groups.

We found significant correlations between gut flora genera and various metabolites in the M and T groups using Spearman’s correlation analysis (Figure 8), where PGE2 was significantly correlated (*p* < 0.05) with all 29 gut flora genera, including Brachybacterium, *Christensenella*, *Streptococcus*, and *Butyricicoccus*, which were significantly associated with the production pathway of PGE2. The area under the curve for metabolites (AUC = 0.889) and the area under the curve for gut flora (AUC = 0.639) by subject-operating curve (ROC) analysis (Supplementary Figure S7) indicated that intestinal metabolites had a higher predictive value than gut flora in the M and T groups. We also compared the flora with a significant difference in PGE2 levels at the genus level in the T, M, and C groups. We found that *Vagococcus*, *Lawsonia*, *Christensenella*, unidentified_*Erysipelotrichaceae*, and unidentified_ *Coriobacteriaceae* were duplicated in groups T and M, suggesting that these five species may be unique to the PGE2 metabolic pathway induced by arecoline.

## 4 Discussion

The prevalence of NAFLD has gradually increased from 5.5% in the past to 11% in 2008, and the proportion of chronic liver disease has also increased from 47% to 75% (Younossi et al., 2011). Treatment strategies include lifestyle interventions (Houttu et al., 2021), drugs (Sanyal et al., 2010) and bariatric surgery (Chavez-Tapia et al., 2010). Vitamin E is the main drug used in patients with NASH and non-comorbid diabetes (Joo et al., 2020). However, betel nut alkaloids reverse NAFLD in rats at therapeutic doses. These results were confirmed by hematological, 16S rRNA, and metabolomic analyses. This may be due to the following reasons: the composition of betel nut commodities is complex and includes not only arecoline but also other components (Tilakaratne et al., 2006); long-term chewing of betel nuts could be associated with toxic side effects, whereas its short-term use may have therapeutic effects without necessarily producing side effects, such as carcinogenesis. Betel nuts have been used for the treatment of parasitic infections and uterine bleeding and as antibacterial agents (Aron-Wisnewsky et al., 2020), but the pharmacological effects of betel nuts in other applications are not well understood. The present study demonstrated its potential therapeutic effect on NAFLD in rats.

An important mechanism in the pathogenesis of NAFLD is the alteration of intestinal flora, including changes in the abundance and diversity of the intestinal microbiota at different stages of the disease (Chiu et al., 2017). In NAFLD, a decrease in the phylum *Bacteroides* has been reported at the bacterial phylum level, whereas the levels of the thick-walled phylum and the phylum Aspergillus increased. Enterobacteriaceae has been reported to increase at the bacterial phylum level, whereas Pronobacteriaceae and Rumenococcaceae have decreased. At the bacterial genus level, *Escherichia coli*, *Doxorubia*, and *Gastroptera* increased, whereas anaerobes, *C. faecalis*, *E. fungalis*, *E. faecalis*, and *Prevotella* decreased (Chiu et al., 2017). Furthermore, transferring the gut microbiota from patients with NASH to germ-free mice while feeding the animals a high-fat diet (HFD) resulted in a substantial increase in ALT, AST, and inflammatory markers, along with hepatic steatosis and liver inflammation, whereas the germ-free mice fed HFD had only lipid accumulation and mild liver inflammation (Hoyles et al., 2018). In contrast, transplantation of microorganisms from patients with NAFLD into the gut of germ-free mice resulted in hepatic steatosis and altered gut microbiota characteristics (Vacca et al., 2020). However, we observed an increase in the abundance of flora in the treated group compared to that in the model group after the use of arecoline, indicating that arecoline partially restored flora diversity in NAFLD. As we tested the results of the medium-concentration group, we could not reflect the situation of the high-concentration group, and whether their flora diversity was positively correlated with arecoline concentration needs to be further investigated. However, histopathology showed that the effect was correlated with arecoline concentration.

This study showed that arecoline affects the intestinal flora. Lachnospiraceae, Desulfovibrionaceae, Deltaproteobacteria, and Actinobacteria changed after arecoline treatment, where the first three were increased, whereas Actinobacteria decreased. Lachnospiraceae belongs to the thick-walled bacteria and the core intestinal flora, and has been shown to be a probiotic of the intestine given its ability to produce butyrate, a beneficial metabolite of the intestine (Elamin et al., 2013). The protective effect of butyrate was achieved through the activation of AMP-activated protein kinase (AMPK) in the monomolecular layer of Caco-2 cells. AMPK regulates the synthesis of glucose, fatty acids, and proteins. The assembly of tight junctions between epithelial cells is impaired when AMPK activity is reduced. Butyric acid protects the intestinal mucosal barrier by increasing AMPK activity and accelerating tight junction assembly between epithelial cells (Sun et al., 2018) and substantially reduces HFD-induced NAFLD in rats, where the reduction in triacylglycerol content is associated with significant activation of PPAR-α (Yun et al., 2019). The current study showed that Desulfovibrionaceae was associated with NAFLD. The reduction in Desulfovibrionaceae was associated with the lean NAFLD group but not with obese NAFLD (Huang et al., 2016), which is consistent with the results of this experiment. However, there have been few studies on Deltaproteobacteria and NAFLD, and future studies are required.

In this study, arecoline lowered blood lipid levels, with effects on TG, TC, LDL-C, and HDL-C levels. The addition of betel nut extract to the daily diet of rats can reduce triglyceride levels, mainly by reducing triglyceride absorption (Marra and Svegliati-Baroni, 2018). Arecoline can improve hepatic glucose metabolism disorders in rats with type 2 diabetes through “exogenous compound receptors, including sexual androstane receptors (CAR) and pregnane X receptors (PXR).” G6Pase gene expression decreases TNF-α and IL-6 expression and increases hepatic insulin sensitivity in type 2 diabetic rats (Borglum et al., 1999; Yang et al., 2019). The findings of this study are consistent with those of previous studies, suggesting that arecoline is a potential hypolipidemic agent. The present study showed that the therapeutic effect are related to arecoline with different concentrations, it seems the high dose group had best effects than others, which still has not been proved by previous studies, further studies are required in to verify these results.

The present study showed that prostaglandin E2 levels were significantly different among the three groups; they were elevated in the model group and decreased in healthy rats. Moreover, PGE2 levels decreased after arecoline treatment, indicating that arecoline could reduce PGE2 levels in NAFLD rats. Combined with the channel prediction analysis, it was found that PGE2 may act in a serotonergic manner. The most effective pathways for NAFLD treatment include arachidonic acid metabolism, synapses, regulation of lipolysis in adipocytes, neuroactive ligand-receptor interactions, and arachidonic acid metabolism. PGE2 has a strong lipolytic effect in adipocytes, which is mediated by the EP3 receptor (Yu et al., 2006). COX-2 expression is upregulated in NASH and can act as a proinflammatory mediator of the metabolic form of steatohepatitis. In MCD diet-induced steatohepatitis, the induction of COX-2 is associated with NF-κB activation and upregulation of TNF-α, IL-6, and ICAM-1. Pharmacological inhibition of COX-2 activity attenuates experimental steatohepatitis (Yu et al., 2006). Green tea protects against liver injury during NASH by reducing COX-2-mediated PGE2 production. Hepatic COX-2 activity and PGE2 concentrations also correlate with serum ALT and hepatic NF-κB (Eeckhaut et al., 2013), suggesting that COX-2/PGE2 is an important pathway in the development of NAFLD and that arecoline protects against hepatic injury through the PGE2/arachidonic acid metabolism pathway to achieve therapeutic effects in NAFLD.

Our study showed that the regulatory effects of PGE2 are associated with *Butyricicoccus*, *Christensenella*, and *Coriobacteriaceae. Butyricicoccus* is a butyric acid-producing bacterium that is reduced in patients with inflammatory bowel disease and is correlated with its severity. Oral administration of *Butyricicoccus pullicaecorum* can reduce colitis and augment epithelial barrier function (Cox et al., 2009). *Butyricicoccus* can produce short-chain fatty acids (SCFA), and butyric acid can induce PGE2 production by activating GPR43, alleviating the inflammatory response caused by LPS (Pascal et al., 2017). *Christensenella* belongs to the thick-walled phylum and has been shown to be associated with a variety of diseases, including Crohn’s disease (Pascal et al., 2017), ulcerative colitis (Pittayanon et al., 2019), and irritable bowel syndrome (Alemán et al., 2018). In addition, *Christensenellaceae* have been shown to substantially increase during weight loss, suggesting a close relationship between this bacterium and the regulation of energy metabolism in the intestinal ecosystem (Yi et al., 2021). *Coriobacteriaceae* has been shown to be associated with impairment of liver function owing to air pollution in patients with schizophrenia, with the main pathways being “NO2/*Coriobacteriales*/GGT” and “NO2/*Coriobacteriales*/GPT” pathways (Liu et al., 2018). *Coriobacteriaceae* has also been shown to partially improve Roux-en-Y gastric bypass to alleviate the dysbiosis of type 2 diabetes, which is beneficial to intestinal flora (Horai et al., 2010). In conclusion, arecoline may regulate the COX-2/PGE2 pathway through multiple intestinal flora and exert therapeutic effects in NAFLD.

Although we demonstrated that arecoline can modulate intestinal flora and metabolites and has potential therapeutic effects on NAFLD, we did not measure intestinal flora and intestinal metabolites at low and high concentrations of arecoline for the treatment of NAFLD. In addition, its specific pathway through PGE2 for the treatment of NAFLD needs to be verified at the cellular and histological levels. Its medicinal value can be explored further through clinical observational and pilot studies.

## 5 Conclusion

Arecoline has a hypolipidemic effect and can regulate the intestinal microflora, which can be used to treat NAFLD through the *Butyricicoccus*/*Christensenella*/*Coriobacteriaceae*-COX 2/PGE2 pathway. Although betel nut alkaloids are currently known carcinogens, they still have some medicinal value, and fully exploiting their potential medicinal value can provide new potential drugs and targets for the treatment of NAFLD.

## Data Availability

The data presented in the study are deposited in the figshare repository, accession number 10.6084/m9.figshare.22285540.

## Supplementary material

Supplementary Figure S1
**(A)** Gross liver map of C vs. M group; **(B)** Lipid levels in C versus M group; **(C)** Gross HE staining of livers in C versus M group.

Supplementary Figure S2
**(A)** Histogram of top20 species composition at the genus level; **(B):** histogram of top20 species composition at the phylum level.

Supplementary Figure S3
**(A):** Genus-level species composition heat map for species clustering; **(B)** genus-level species composition heat map for biclustering.

Supplementary Figure S4
**(A)** Seed network diagram of dominants with grouped abundance pie charts; **(B)** KEGG metabolic pathway differing between groups; **(C)** MetaCyc metabolic pathway differing between groups; **(D)** LEU-DEG2-PWY:L-leucine degradation I differential species composition diagram.

Supplementary Figure S5
**(A)** PLS-DA score plot (T vs. M vs. C); **(B)** Hierarchical cluster analysis of overall metabolite heat map (positive model); **(C)** Venn diagram of differential metabolites (T vs. M vs. C); **(D)** Statistical histogram of differential metabolites (positive model).

Supplementary Figure S6
**(A)** Heat map of differential metabolite hierarchical clustering (positive model) (T vs. M); **(B)** Volcano plot of differential metabolites (positive model) (T vs. M).

Supplementary Figure S7 Metabolites combined with species (genus) ROC curves (T vs. M).
