# Supplementary figures and images for: Gut metabolomics and 16S rRNA sequencing analysis of the effects of arecoline on non-alcoholic fatty liver disease in rats
Source: Front Pharmacol. 2023 Mar 27;14:1132026. doi: 10.3389/fphar.2023.1132026 (PMC10083296; doi:10.3389/fphar.2023.1132026)

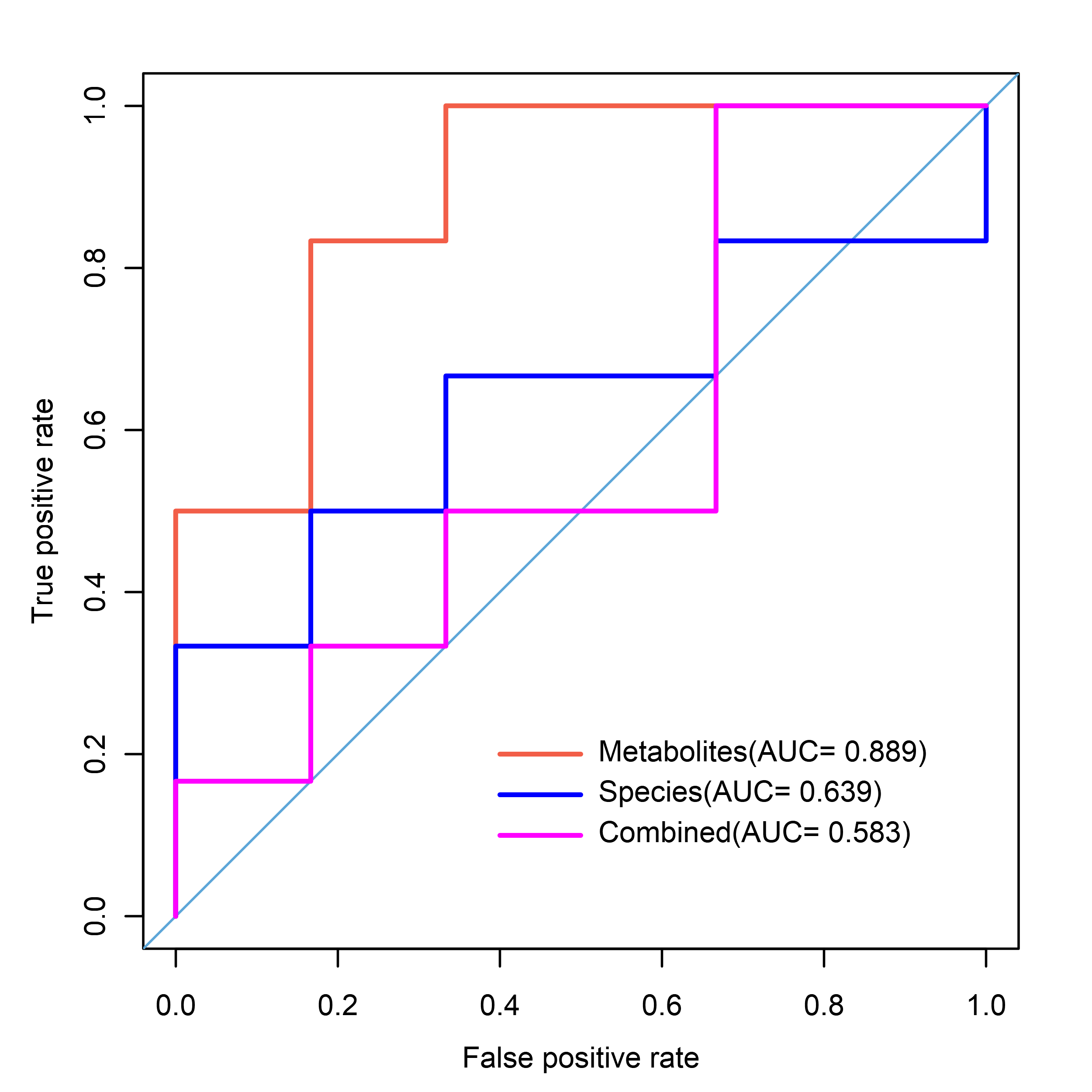

Supplement: Supplementary file 6 [file Image7.TIF]
